# An AIE-active probe acts as novel tool for rapid and accurate quantitative antifungal susceptibility assessment

**DOI:** 10.3389/fmicb.2025.1566846

**Published:** 2025-03-27

**Authors:** Fen Zheng, Xiaoxue Ge, Yajuan Guan, Qixian Zhou, Renren Shi, Jiayi Zhao, Bo Situ, Jing Zhang, Yongyu Rui

**Affiliations:** ^1^Department of Laboratory Medicine, Nanfang Hospital, Southern Medical University, Guangzhou, China; ^2^Department of Laboratory Medicine, The Affiliated Foshan Women and Children Hospital, Guangdong Medical University, Foshan, China

**Keywords:** antifungal susceptibility assessment, aggregation-induced emission, TBP-2, *Candida albicans*, fungal infection

## Abstract

**Objectives:**

Common antifungal susceptibility testing methods are often time-consuming and subject to interpretation bias in endpoint determination, making them inadequate for clinical applications. We aim to develop a rapid and accurate quantitative method for routine antifungal susceptibility testing in diagnostic laboratories by employing the aggregation-induced emission (AIE) luminogen TBP-2.

**Methods:**

The AIE luminogen TBP-2 with two positive charges was introduced to develop an antifungal susceptibility testing protocol based on the reference broth microdilution (BMD) method. The minimum inhibitory concentration of different drugs against *Candida* was determined by detecting changes in fluorescence intensity. A total of 76 clinical isolates of *Candida albicans* (*C. albicans*) were collected to evaluate the performance of the platform. The results obtained by the TBP-2-based method were compared with those obtained by the reference BMD.

**Results:**

The TBP-2-based method enables endpoint determination by detecting fluorescence intensity after a co-incubation period of 8 h with *C. albicans* in drugs. The excellent essential agreement between the TBP-2-based test and BMD among 76 clinical isolates was observed for all the four drugs. The categorical agreement between two methods was 100% for amphotericin B and 5-flucytosine, 96.1% for fluconazole and 97.4% for voriconazole. Only minor errors were found in fluconazole and voriconazole, at 3.9 and 2.6%, respectively, with no errors found in very major errors and major errors.

**Conclusion:**

The TBP-2-based method provides rapid and accurate quantifiable endpoints, aiding in the timely selection of appropriate antifungal therapy, and offering opportunities for automation and widespread application.

## 1 Introduction

The incidence of invasive fungal infections caused by *Candida* species continues to rise, primarily attributed to the increased populations of immunocompromised patients and those undergoing invasive procedures (Brown et al., 2012; Fisher et al., 2018; Maschmeyer, 2006; Tortorano et al., 2012; van de Veerdonk et al., 2010; Zatta et al., 2020). *Candida* infection has emerged as a significant component of nosocomial infections, with a mortality rate for candidemia reaching as high as 40–60%, posing a serious threat to patients’ life and health (Gudlaugsson et al., 2003; Lee et al., 2021; Li et al., 2016; Lin et al., 2018; Qiao et al., 2023; van de Veerdonk et al., 2010). Like bacteria, *Candida* is also experiencing drug resistance due to the wide application of antifungal drugs (Hokken et al., 2019; Lee et al., 2021; Pintye et al., 2024; Sharma et al., 2024). As a result, the establishment of a reliable and rapid method to determine the susceptibility of isolates to antifungal drugs would therefore be beneficial in the treatment of patients with invasive fungal disease.

While methods for *in vitro* susceptibility testing were developed as new antifungal drugs were identified, the reproducibility of these assays among laboratories was varied. The Clinical and Laboratory Standards Institute [CLSI; formerly the National Committee for Clinical Laboratory Standards (NCCLS)] has developed and published an approved reference method for broth microdilution testing (BMD, CLSI document M27-A4) of *Candida* species (Clinical Laboratory Standards Institute, 2017). Application of the reference BMD method has minimized the variation among laboratories. However, this method is not only time-consuming, but also subjective in the interpretation of endpoints because of the trailing-growth phenomenon (Kim and Lee, 2010). Based on the reference method, several commercial methods have been developed and used by clinical microbiology laboratories, such as Sensititre YeastOne assay and ATB FUNGUS 3. Unfortunately, although these methods simplify the procedure and optimize the endpoint interpretation, they are far from satisfactory, mainly because they require a long test time to observe the effects of candidate drugs on the growth of *Candida*. Moreover, these methods still fail to completely address the challenge of subjective interpretation of the endpoint. In order to reduce the test time, the *Candida* susceptibility tests based on flow cytometry by detecting the biochemical changes in *Candida* cells after a short period of drug treatment have been carried out, but its clinical application is limited mainly due to the low throughput and the requirement for expensive supporting equipment (Gokahmetoglu et al., 2003; Joung et al., 2007; Rudensky et al., 2005). Therefore, a rapid, accurate quantitative, high-throughput, and convenient method is required for routine antifungal susceptibility testing of clinical isolates in diagnostic laboratories.

Since 2001, a unique phenomenon of aggregation-induced emission (AIE) has been observed in propeller-shaped molecules, which emit faintly in their solutions but fluoresce intensely when aggregated (Luo et al., 2001). Through systematic studies, restriction of intramolecular motion (RIM) was identified as the main cause for the AIE effect. This phenomenon is the opposite of the traditional aggregation-caused quenching. Molecules with AIE properties offer significant advantages over traditional fluorescent probes in biological detection (Zhao et al., 2020)^.^ Their unique ability to dramatically increase fluorescence upon aggregation makes them ideal for fluorescence amplification-based quantitative detection. Additionally, AIE molecules can bind to biological macromolecules more efficiently than traditional probes, resulting in much higher fluorescence intensity. These features contribute to superior biological detection sensitivity and facilitate more reliable fluorescence measurements. The advantages of AIE-based materials have led to enhanced development in various biological applications, including cell (Situ et al., 2020) and bacteria imaging (Broeren et al., 2013; Maruthi and Kalangi, 2021), tissue (Wang et al., 2014) and tumor visualization (Qin et al., 2012; Zhang et al., 2015), therapy (He et al., 2019; Shi et al., 2020a), and antibiotics susceptibility testing (Ge et al., 2022; Zhu et al., 2011). The fluorescence turn-on characteristics of the AIE luminogens upon binding to targets make them excellent candidates as fluorescence sensors and inspire further exploration of their potentials in *Candida* susceptibility evaluation (Zhao et al., 2020).

The present study aimed to develop a rapid, accurate quantitative and high-throughput method with the aid of an AIE luminogen 4-(7-(4-(diphenylamino)phenyl)benzo[c][1,2,5]thiadiazol-4-yl) − 1-(3-(trimethylammonio)propyl)pyridin-1-ium bromide (TBP-2) for routine antifungal susceptibility testing of clinical isolates in diagnostic laboratories.

## 2 Materials and methods

### 2.1 Materials and strains

The utilized AIE-active probe, TBP-2, was synthesized following the procedures reported previously (Shi et al., 2020a,b). Dimethyl sulfoxide (DMSO), 2,2,6,6-tetramethyl-4-piperidinol (TEMP) and 9,10-anthracenediyl-bis(methylene) dimalonic acid (ABDA) were from Sigma-Aldrich. 1-Bromoethane, (3-bromopropyl)trimethylammonium bromide, acetonitrile, and diethyl ether were purchased from J&K scientific. All reagents with analytical purity and were used without further purification. A stock solution of TBP-2 in DMSO with a concentration of 10 × 10^−3^ M was prepared and stocked in the 4°C fridge. RPMI 1640 medium (powder, with L-glutamine, without bicarbonate) was purchased from Sigma. Antifungal drugs include fluconazole (FCA), voriconazole (VRC), 5-flucytosine (5-FC), and amphotericin B (AMB) powders were purchased from the National Institutes for Food and Drug Control (Beijing, China). Morpholinepropanesulfonic acid (MOPS) was purchased from MPbio. Sabouraud dextrose agar (SDA) were purchased from Zhengzhou Antu Biological Co., Ltd. Common consumables such as centrifuge tubes, 96-well plates, and pipette tips were purchased from NEST and Axygen. *Candida albicans* (*C. albicans*) ATCC 90028, *Candida krusei* (*C. krusei*) ATCC6258 and *Candida parapsilosis* (*C. parapsilosis*) ATCC22019 were purchased from American Type Culture Collection (ATCC). All the clinical isolates were collected and used with approval of the Ethics Committee of Foshan Women and Children Health Hospital (No: FSFY-MEC-2024-095).

### 2.2 RPMI 1640 broth medium

Powdered RPMI 1640 medium 10.4 g, 34.53 g of MOPS, and 18 g of glucose; are dissolved in 900 mL of distilled water while stirring until fully dissolved. The pH is adjusted to 7.0 ± 0.05 at 25°C using 1 mol/L sodium hydroxide, and the medium is brought up to 1,000 mL with additional water. Then the medium is filtered and stored at 4°C for later use.

### 2.3 Drug diluent

The storage solution of 200 times final concentration was prepared with DMSO as solvent and RPMI 1640 broth medium as diluent, and then the storage solution was diluted with RPMI 1640 broth at 1:100 to obtain working solution of twofold final concentration. The final drugs concentration ranges were 0.125–16 μg/mL for AMB, 0.25–16 μg/mL for 5-FC, 0.25 to 128 μg/mL for FCA, and 0.03–8 μg/mL for VRC.

### 2.4 *Candida* suspension

Clinical isolates were collected and identified by mass spectrometry. Before use, each isolate was passaged at least twice on SDA to ensure its purity and viability. *C. albicans* ATCC 90028 was also used for quality assessment of susceptibility testing. Several single colonies were suspended in RPMI 1640 broth medium and adjusted to the concentration required for BMD method or TBP-2-based method. The concentration of *Candida* suspension was counted using an improved Neubauer counting chamber under optical microscope. The count was repeated three times to take the average value.

### 2.5 *Candida* imaging

To capture fluorescence images, *Candida* suspension was co-incubated with 10 μmol/L TBP-2 at 35°C for 10 min, then 5 μL of the *candida* resuspension was dropped onto a piece of glass slide and covered by a coverslip. The images were collected using a confocal laser scanning microscope (LSM900, Carl Zeiss, Germany).

### 2.6 Correlation curve of concentration and fluorescence intensity

A series twofold RPMI 1640 broth medium dilutions of *C. albican* ATCC90028 and TBP-2 were dispensed into 96-well plate. The concentration of *Candida* in the mixtures ranged from 6.25 × 10^5^ to 1.6 × 10^8^ cells/mL, and the final concentration of TBP-2 was 10 μmol/L. The mixed solutions were then incubated at 35°C for 10 min, followed by measuring of fluorescence intensity employing the TECAN SPARK Multifunctional Microplate Reader [Tecan, Tecan (Shanghai) Trading Co., Ltd] using 488 and 679 nm as excitation and emission wavelengths, respectively.

### 2.7 Reference BMD method

The reference BMD tests were performed according to the CLSI M27-A4 guidelines (Clinical Laboratory Standards Institute, 2017). The suspension of *C. albicans* ATCC 90028 or clinical isolates was diluted in RPMI 1640 medium to yield an inoculum concentration of approximately 1 × 10^3^ to 5 × 10^3^cells/mL. One hundred microliters per well was inoculated into wells containing dilutions of each drug with the same volume. The mixtures were incubated at 35°C for 24 h. Meanwhile, the blank control with only broth medium and the growth control with only *candida* suspension were set up for each preparation. Each test was repeated three times, and the final result was chosen if the outcomes varied.

The minimal inhibitory concentration (MIC) endpoint was scored by comparing the growth in each well with that in the growth control well. The MIC values of the azole antifungals and 5-FC were defined as the minimum drug concentration at which visual growth was determined to be 50% relative to that of the growth control, known as MIC_50_. The MIC of AMB was defined as the lowest drug concentration at which there was 100% inhibition of growth compared with the growth control, referred to as MIC_100_.

### 2.8 TBP-2-based method

The *C. albicans* RPMI 1640 broth suspension with a concentration of 1 × 10^6^ to 5 × 10^6^ cells/mL at a volume of 100 μL was added to a 96-well plate containing equal volume antifungal drugs working solution, and the mixtures were incubated at 35°C for 8 h. Two wells were designated as negative control and positive control without any drug. The negative control was filled with RPMI 1640 broth culture medium without *C. albicans*, while the positive control was filled with 1,640 broth culture medium containing *C. albicans*. The final drugs concentration ranges were 0.125–16 μg/mL for AMB, 0.25–16 μg/mL for 5-FC, 0.25–128 μg/mL for FCA, and 0.03–8 μg/mL for VRC. The incubation was suspended, and 90 μL was added to a new 96-well plate. The unincubated initial concentration of *Candida* RPMI 1640 broth suspension was also added to the 96-well plate as a blank control. Then, 10 μL of 100 μmol/L TBP-2 was added, mixed well, and incubated at 35°C for 10 min. The final concentration of TBP-2 in the wells was 10 μmol/L. After an 8-h incubation, the fluorescence intensity of each well was detected using the TECAN SPARK Multifunctional Microplate Reader. The intensity was measured three times to obtain the average value. The fluorescence of the growth in the drug-free control was defined as 100%, and the drug-exposed wells were scaled to this value. The relative change in fluorescence intensity was obtained by the formula *ΔI* = (*I_g_ - I*)/(*I_g_-I_b_*). I represents the fluorescence intensity of the test well, *I_g_* is the fluorescence intensity of drug-free growth control, and the fluorescence intensity of the blank control well containing initial concentration of *Candida* suspension is *I_b_*. The interpretation endpoint for 5-FC, FCA, and VRC is the MIC_50_, defined as the minimum drug concentration that decreases the fluorescence intensity by 50% compared to the drug-free growth control. For AMB, the interpretation endpoint is the MIC_90_, the minimum drug concentration that reduces the fluorescence intensity by 90% compared to the drug-free growth control. The brief scheme of this method is shown in Supplementary material S1.

### 2.9 Analysis of susceptibility testing results

The MIC values obtained with the TBP-2-based test were compared with those obtained with the reference BMD method. The clinical breakpoints (CBPs) were used to obtain the categorical agreement (CA; susceptibility results that fall within the same interpretive category) between the TBP-2-based and reference MIC values. CBPs for FCA (S, ≤ 2 μg/mL; SDD, 4 μg/mL; R, ≥8 μg/mL) and VRC (S, ≤0.12 μg/mL; I, 0.25–0.5 μg/mL; R, ≥1 μg/mL) came from CLSI, while those for AMB (S, ≤1 μg/mL; R, >1 μg/mL) and ITR (S, ≤ 0.06 μg/mL; R, >0.06 μg/mL), due to the absence of CLSI breakpoints, came from European Committee on Antimicrobial susceptibility Testing (EUCAST). The antifungal agent 5-FC was not judged due to the absence of CBPs. Very major errors (VME) were identified when the reference MIC categorized an isolate as resistant but the TBP-2-based MIC categorized it as susceptible (falsely susceptible). Major errors (ME) were identified when the reference method categorized the isolate as susceptible but the TBP-2-based method categorized it as resistant. Minor errors were determined when the result of one of the test methods was either susceptible or resistant and that of the other was SDD or Intermediate. In cases of discrepant results, testing of both methods (the TBP-2-based method and reference BMD) were repeated and the results for the second runs were accepted as the final results. According to the CLSI document (M23-Ed6), the acceptable rates of error of the methodology tested when compared with the gold standard were the following: <1.5% of VME, <3% of ME and < 5% of Minor errors (Clinical Laboratory Standards Institute, 2023). Essential agreement (EA) was considered when results presented by TBP-2-based and BMD methods exhibited agreement within ±1 two-fold dilution (Clinical Laboratory Standards Institute, 2023). The target intermethod correlation should be greater than 90% according to the CLSI document (M23-Ed6) (Clinical Laboratory Standards Institute, 2023). The Kappa coefficient statistic was also used to evaluate assay agreement, where values above 0.8 represented almost perfect agreement (McHugh, 2012).

## 3 Results

### 3.1 Synthesis and characterization of TBP-2

**Figure 1:**
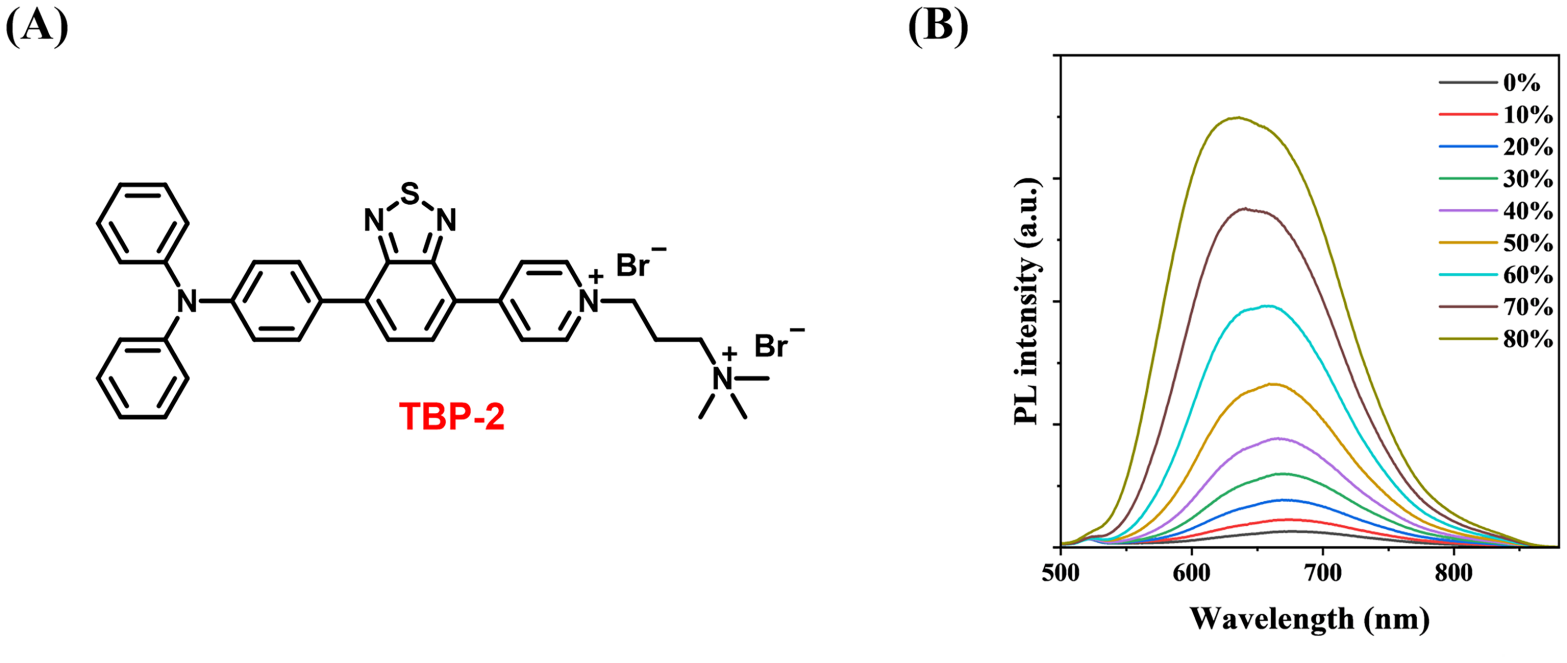
**(A)** Chemical structure of TBP-2 (yield 65%, purity 95%). **(B)** Change in fluorescence intensity of TBP-2 in DMSO/toluene mixtures with different toluene fractions (*f_t_*). Conditions: *λ*_ex_ = 488 nm.

TBP was facilely synthesized by two stepwise Suzuki couplings followed by its reaction with 3-bromopropyl trimethylammonium bromide to yield TBP-2 (200 mg, yield 65%, Figure 1A). ^1^H NMR (400 MHz, MeOD-d_4_), *δ* (TMS, ppm): 9.17–9.15 (2H, d), 9.07–9.05 (2H, d), 8.52–8.50 (1H, d), 8.10–8.07 (3H, dd), 7.39–7.35 (4H, t), 7.20–7.13 (8H, m), 4.83–4.80 (2H, t), 3.67–3.63 (2H, m), 3.26 (9H, s), 2.69 (2H, m). ^13^C NMR (400 MHz, MeOD-d_4_), δ (TMS, ppm): 153.86, 153.52, 152.78, 149.33, 147.16, 144.25, 138.01, 132.28, 130.37, 129.24, 126.63, 126.28, 125.05, 123.74, 121.49, 62.49, 57.10, 52.60, 24.73. Purity: 95%. The structure of TBP-2 was characterized by NMR. Detailed data were provided in Supplementary Figures S2, S3. The amines in TBP-2 endow it two positive charges. The positively charged amines endow TBP-2 with the hydrophilicity, allowing it to dissolve in water.

To examine the AIE feature of TBP-2, the photoluminescence (PL) intensity of TBP-2 was measured in the mixed solvents of DMSO/toluene with gradually increasing the fractions of toluene. Upon the addition of toluene, a poor solvent for TBP-2, into its DMSO solution, the fluorescence gradually intensifies as the toluene fraction (*f_t_*) rises from 0 to 80%, due to the formation of aggregates (Figure 1B). This result verifies the AIE property of TBP-2.

### 3.2 Fluorescence imaging of *Candidas*

**Figure 2:**
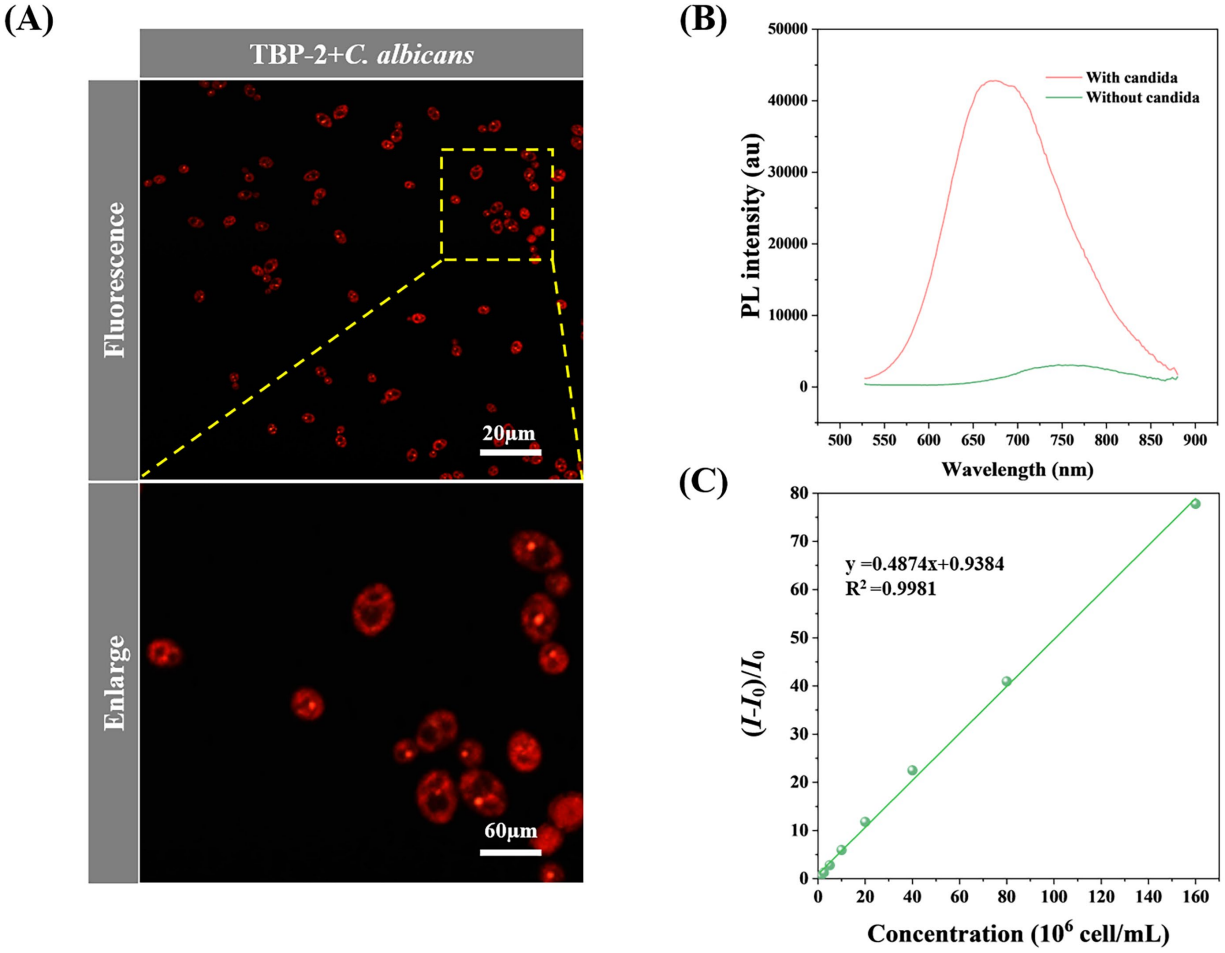
**(A)** Confocal laser scanning microscope (CLSM) image of *C. albicans* incubated with 10 μmol/L of TBP-2 for 10 min. **(B)** PL spectra of TBP-2 with/without 10^8^ cell/mL of *C. albicans*. **(C)** Change in fluorescence intensity of TBP-2 with the concentration of *C. albicans* in RPMI 1640 broth medium. *I*: fluorescence intensity of *candida* suspensions, *I_0_*: fluorescence intensity of RPMI 1640 broth medium. Condition: *λex* = 488 nm.

The standard strain *C. albicans* ATCC900298 was incubated with TBP-2 and cultured at 35°C for 10 min before imaging. As shown in Figure 2A, *C*. *albicans* are imaged clearly under the fluorescence microscope. The standard strains ATCC22019 *C. parapsilosis* and ATCC6258 *C. krusei*, representing non-*C. albicans* species, were co-incubated with TBP-2 and stained. The staining images consistently demonstrated that non-*C. albicans* species exhibited a staining pattern similar to that of *C. albicans* (Supplementary Figure S5). This indicates that the staining effect is reproducible across various *Candida* species and is not dependent on the cell wall structure. To quantify the difference in fluorescence intensity, we measured the PL spectra of the solutions (Figure 2B). The solution of TBP-2 and *C. albicans* at 1.6 × 10^8^ cell/mL displays 75 times higher emission intensity than that of TBP-2 alone.

The fluorescence response of TBP-2 to different concentrations of *C. albicans* were investigated. Interestingly, the fluorescence intensity of TBP-2 takes linear relationship with *C. albicans* concentrations in the range of 6.25 × 10^5^ to 1.60 × 10^8^ cell/mL (Figure 2C). Therefore, *Candida* amount in culture media can be easily identified by measuring the fluorescence intensity of the culture solutions.

### 3.3 Testing of the standard strain ATCC90028

**Figure 3:**
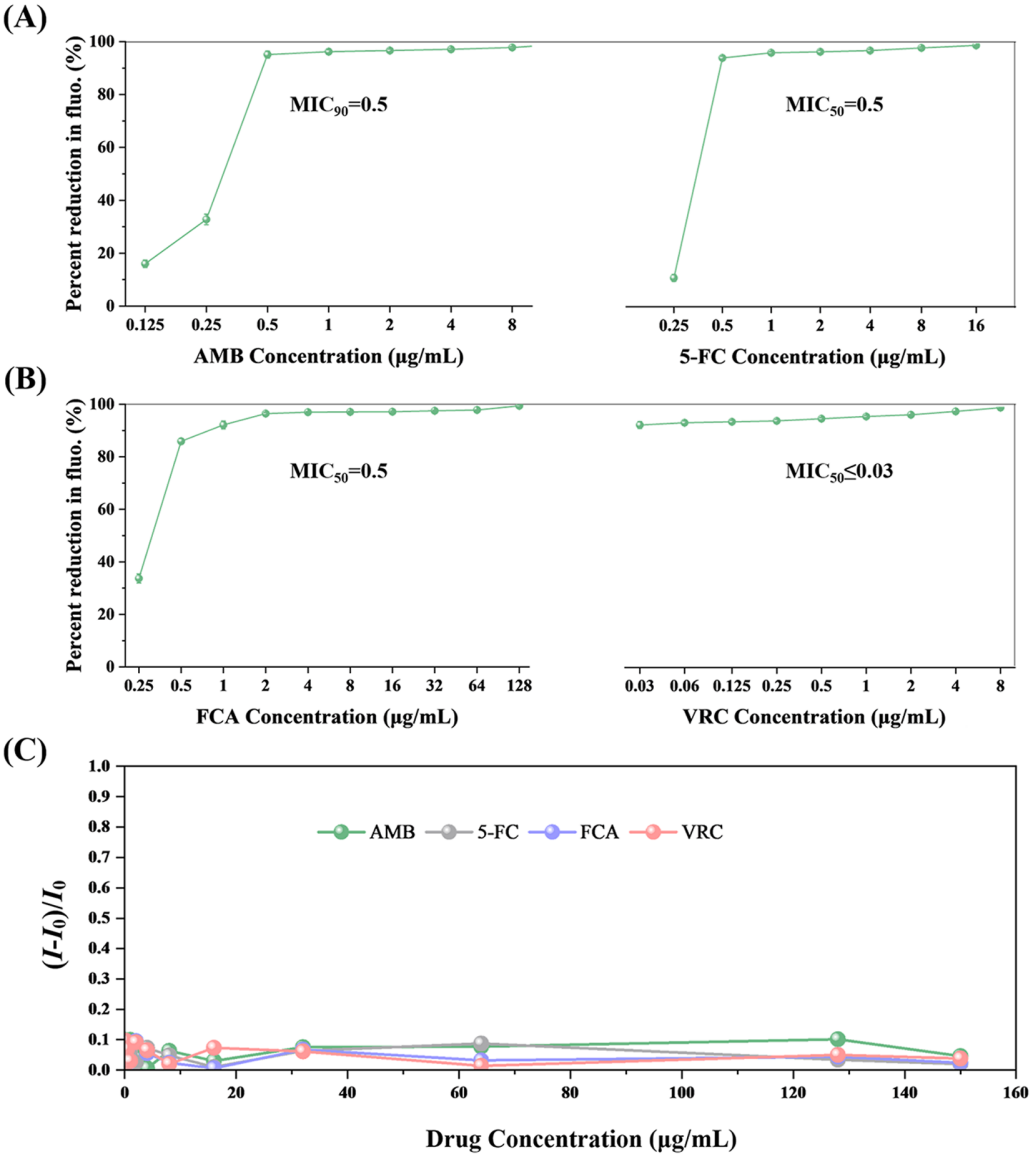
The MIC values of AMB **(A)**, 5-FC **(A)**, FCA **(B)**, VRC **(B)** for *C. albicans* ATCC90028 using the TBP-2-based method. The fluorescence of the growth in the drug-free control is defined as 100%. The error bars represent standard errors. **(C)** Changes in fluorescence intensity of TBP-2 in RPMI 1640 broth medium in the presence of different antifungal drugs. *I_0_*: fluorescence intensity of RPMI 1640 broth medium. Condition: *λ*ex = 488 nm.

The standard strain *C. albicans* ATCC90028 was used to verify the feasibility of the TBP2-based method. After 8 h of incubation, the concentration of *Candida* in the growth control well increased approximately tenfold, reaching about 5 × 10^7^ cells/mL. Both the initial and final concentrations fell within the linear range of 6.25 × 10^5^ to 1.60 × 10^8^ cell/mL. This allowed us to determine the MIC for four antifungal drugs based on changes in fluorescence intensity. The MIC values for the four drugs determined using the TBP-2-based method are presented in Figures 3A,B. In comparison to the BMD method, the TBP-2 method yielded the same MIC values for 5-FC, AMB, and VRC, while the MIC for FCA was one two-fold dilution higher (Table 1). The TBP-2-based method showed good EA and CA with the BMD method when detecting the susceptibility of standard strain ATCC90028 to four antifungal drugs.

**Table 1:** The results of antifungal susceptibility test for *C. albicans* ATCC90028 detected by TBP-2-based method and BMD method.

Antifungal drugs	TBP-2-based method		BMD method	
	MIC^a^ (μg/mL)	Phenotype	MIC^b^ (μg/mL)	Phenotype
5-FC	0.5	NA	0.5	NA
AMB	0.5	S	0.5	S
FCA	0.5	S	0.25	S
VRC	≤0.03	S	≤0.03	S

a The MIC values for 5-FC, FCA, and VRC are determined at MIC_50_, whereas for AMB, the MIC value is determined at MIC_90_.

b The MIC values for 5-FC, FCA, and VRC are determined at MIC_50_, whereas for AMB, the MIC value is determined at MIC_100_.

MIC, minimal inhibitory concentration; S, susceptible; NA, not applicable.

In addition, we investigated how antifungal drugs affect the fluorescence intensities of TBP-2. Figure 3C and Supplementary Table S1 showed that even at concentrations of 150 μg/mL, none of the four antifungal drugs significantly increased emission intensity.

### 3.4 Testing of clinical isolates

A total of 76 strains of *C. albicans* that isolated from clinical specimens were collected to evaluated the performance of the platform. In parallel, reference BMD method were performed for comparison.

**Figure 4:**
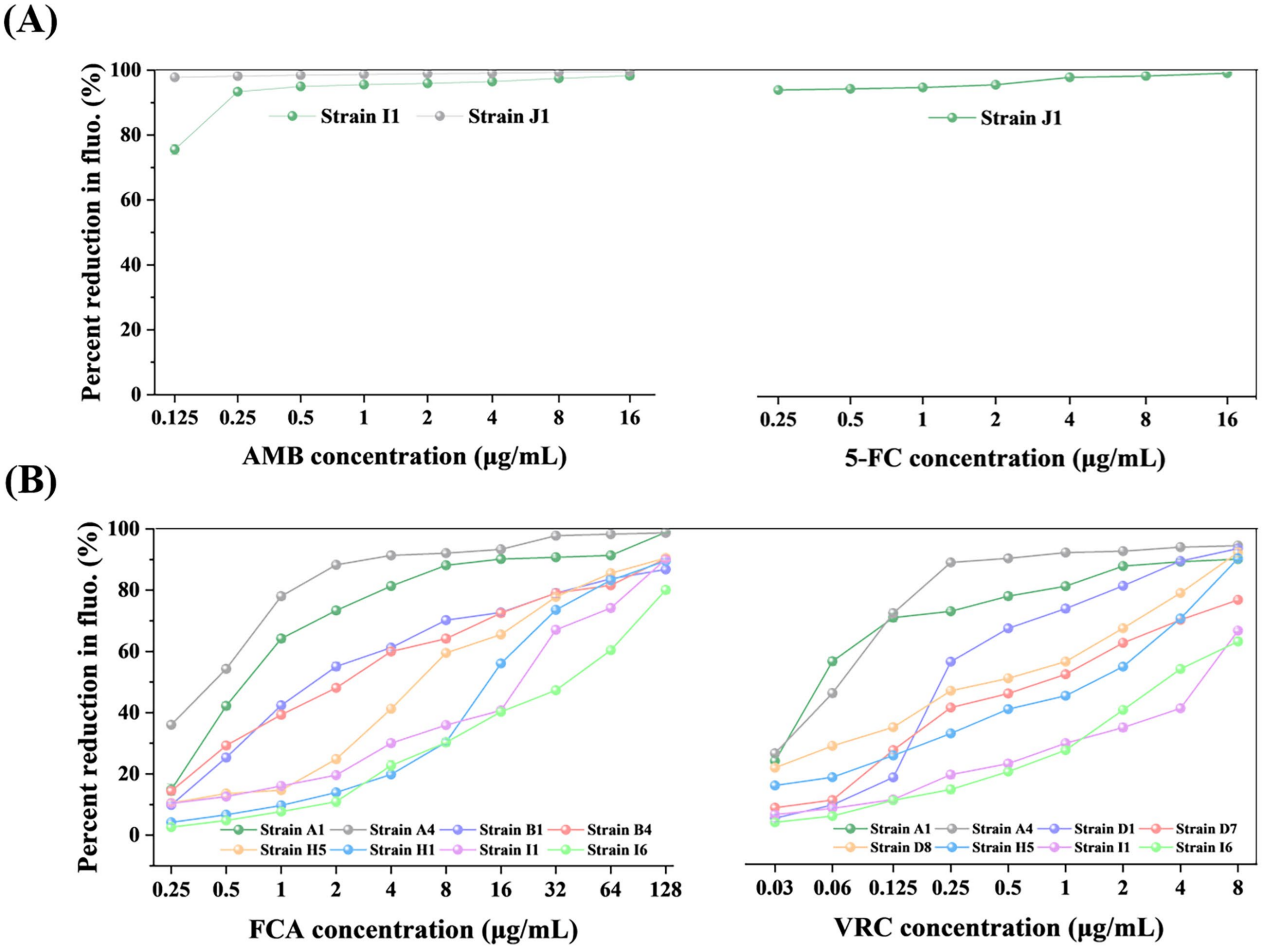
The MIC endpoints of representative clinical isolates for AMB (A), 5-FC **(A)**, FCA **(B),** and VRC **(B)** determined by TBP-2-based method. The results are as follows: For AMB **(A)**, the MIC values for strains J1 and I1 are ≤0.125 and 0.25, respectively, both are considered susceptible; for 5-FC **(A)**, the MIC value for strain J1 is ≤0.25; for FCA **(B)**, the MIC values for strains A4, A1, and B1 are 0.5, 1, and 2, respectively, which are considered susceptible, the MIC value for strain B4 is 4, which is considered susceptible dose-dependent, and the MIC values for strains H5, H1, I1, and I6 are 8, 16, 32, and 64, respectively, which are considered resistant; for VRC **(B)**, the MIC values for strains A1 and A4 are 0.06 and 0.125, respectively, which are considered susceptible, the MIC values for strains D1 and D8 are 0.25 and 0.5, respectively, which are considered Intermediate, and the MIC values for strains D7, H5, I6, and I1 are 1, 2, 4, and 8, respectively, which are considered resistant. The fluorescence of the growth in the drug-free control was defined as 100%, and the drug-exposed wells were scaled to this value. The error bars represent standard error. The unit of MIC is μg/mL.

The MIC_90_ values of the test strains for AMB are ≤0.125 μg/mL for 14 strains and 0.25 μg/mL for 62 strains, all of which are susceptible, according to their reduction of at least 90% in relative fluorescence intensity scaled to the drug-free control (Figure 4A; Table 2). For 5-FC treatment, the MIC_50_ values for 76 strains were all ≤0.25 μg/mL, according to their reduction of at least 50% in relative fluorescence intensity scaled to the drug-free control (Figure 4A). The *C. albicans* isolates chosen covered a broad range of susceptibilities according to their reduction of at least 50% in relative fluorescence intensity scaled to the drug-free control: for FCA, 0.5–64 μg/mL and for VRC, 0.06 to 8 μg/mL (Figure 4B). The MIC values for ATCC 90028 control strain was within the accepted limits.

**Table 2:** Assessment of the consistency of antifungal susceptibility results obtained by the TBP-2-based and BMD method for 76 clinical *Candida* isolates.

Antifungal drugs	Test Method	% of MIC by category (No.)				CA (%)	Kappa coefficient	% errors		
		S	SDD	I	R			% VME	% ME	% Minor
AMB	TBP-2-based	100 (76)	0(0)	0 (0)	0 (0)	100	1	0	0	0
	BMD	100 (76)	0(0)	0 (0)	0 (0)					
5-FC	TBP-2-based	NA	NA	NA	NA	100	1	0	0	0
	BMD	NA	NA	NA	NA					
FCA	TBP-2-based	23.7 (18)	31.6 (24)	NA	44.7 (34)	96.1	0.939	0	0	3.9
	BMD	23.7 (18)	32.9 (25)	NA	43.4 (33)					
VRC	TBP-2-based	28.9 (22)	NA	36.8 (28)	34.2 (26)	97.4	0.960	0	0	2.6
	BMD	30.3 (23)	NA	36.8 (28)	32.9 (25)					

S, susceptible; SDD, susceptible dose dependent (for FCA only); I, Intermediate (for FCA only); R, resistant; CA, categorical agreement; VME, very major error; ME, major error; Minor, minor error; NA, not applicable.

Table 2 summarized the susceptibilities of 76 clinical isolates to AMB, FCA and VRC judged by TBP-2-based and BMD method. The antifungal agent 5-FC was not defined due to the absence of clinical breakpoints. The CA was 100% for AMB and 5-FC, 96.1% for FCA and 97.4% for VRC. For FCA and VRC, which did not achieve 100% for CA, the Kappa coefficients were 0.939 and 0.960, respectively. These values are both close to 1, indicating that the TBP-2-based method and the BMD method exhibit almost perfect agreement. Only minor errors were found in FCA and VRC, at 3.9 and 2.6%, respectively, with no errors found in VEM and ME. For VRC, two minor errors were due to TBP-2-based method producing higher MIC values, and for FCA, three minor errors were observed, two of which were due to TBP-2-based method producing higher MIC values, and one due to TBP-2-based method producing a lower MIC value (Supplementary Table S2). Where MIC endpoints differed between the two methods, for VRC, the interpretive susceptibility category changed for two strains, with one changing from S to I and the other from I to R. For FCA, the interpretive susceptibility category changed for three strains, with one changing from S to SDD, one changing from SDD to S, and another changing from SDD to R (Supplementary Table S2).

**Figure 5:**
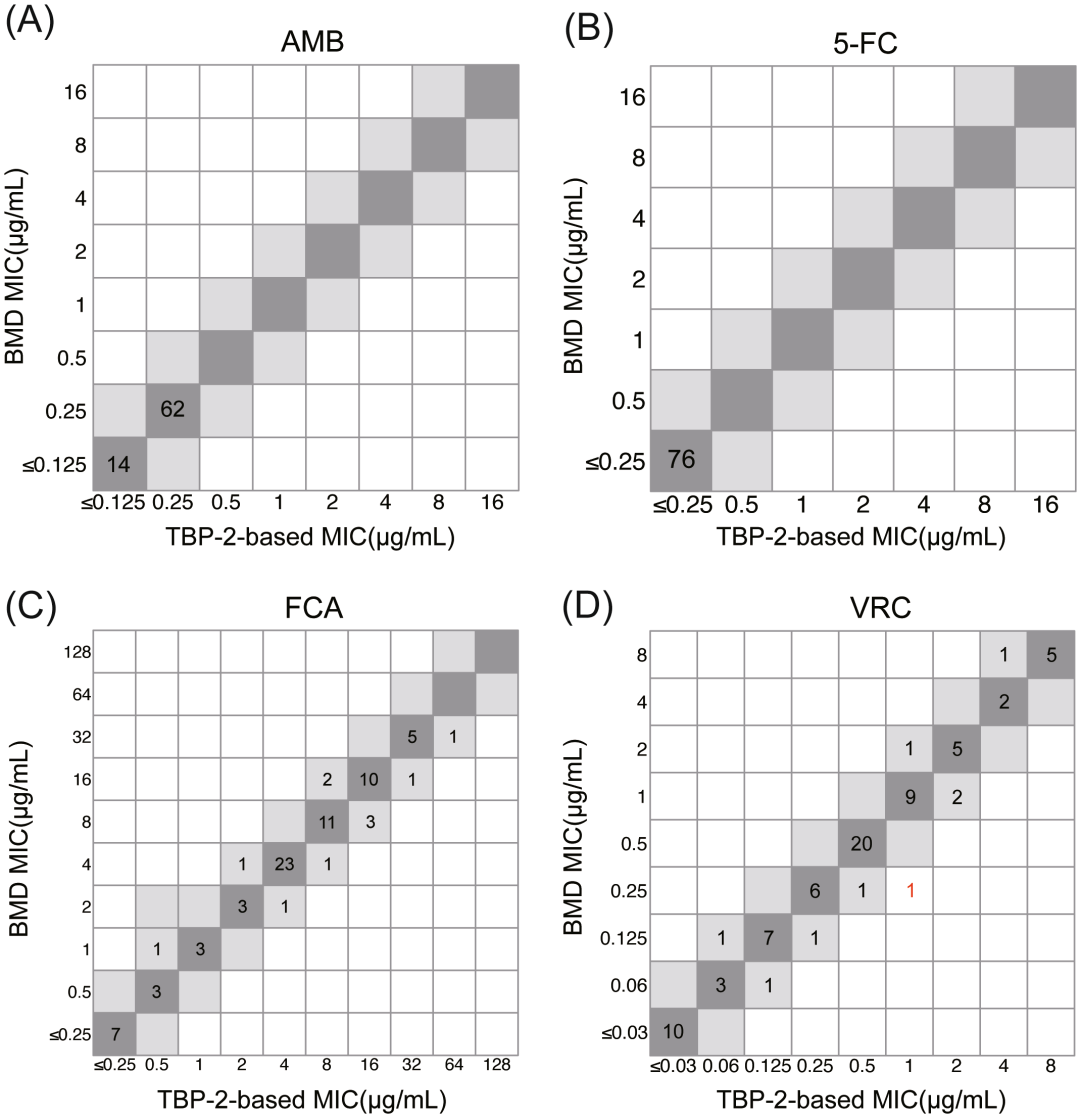
Correlation between TBP-2-based and BMD methods for *C. albicans* against AMB **(A)**, 5-FC **(B)**, FCA **(C)** and VRC **(D)**. MIC values within ±1 two-fold dilution are highlighted in gray. Strains that exceed the EA range are marked in red.

As shown in Table 3 and Figure 5, excellent EA (within 1 dilution) between the TBP-2-based test and reference BMD was observed for AMB, 5-FC, FCA (100%), and VRC (98.7%). The discrepancy in EA of VRC was due to TBP-2-based method producing higher (2 dilutions) MIC.

**Table 3:** Distribution of differences in MIC results between TBP-2-based and BMD for 76 *C. albicans* isolates.

Antifungal drugs	Number of isolates with TBP-2-based MIC values different from reference BMD method (%)							% EA ^a^
	<-2	-2	-1	0	1	2	>+2	
AMB	0 (0)	0 (0)	0 (0)	76 (100)	0 (0)	0 (0)	0 (0)	100
5-FC	0 (0)	0 (0)	0 (0)	76 (100)	0 (0)	0 (0)	0 (0)	100
FCA	0 (0)	0 (0)	4 (5.3)	65 (85.5)	7 (9.2)	0 (0)	0 (0)	100
VRC	0 (0)	0 (0)	3 (3.9)	67 (88.2)	5 (6.6)	1 (1.3)	0 (0)	98.7

a EA of MIC results of the two methods was defined as agreement within ±1 two-fold dilution.

## 4 Discussion

The emerging magnitude of human fungal infections has renewed interest in developing rapid and standardized method for susceptibility testing. The primary challenge with existing susceptibility testing methods, including the BMD reference method, is that they are time-consuming, difficult and subjective when determining the endpoint.

Conventional antifungal susceptibility testing methods primarily rely on culture-based techniques. In addition to the standard reference method BMD, several widely used commercial methods are also included, such as ATB FUNGUS 3, YeastOne, and Vitek 2. ATB FUNGUS 3 is a 32-well strip that contains varying concentrations of five antifungal agents. After inoculation and incubation for 24–48 h, growth is read visually or automatically with the mini Api instrument. Although ATB FUNGUS 3 is operationally simpler than the BMD method, the incubation time has not been shortened, and subjective issues still exist when determining the test endpoint for trailing drugs (Zhang et al., 2014). Sensititre YeastOne assay is a technique similar to the BMD method, but it uses a colorimetric growth agent (alamarBlue), making the result reading more intuitive through color changes. While it is less subjective than the BMD method, interpreting trailing growth in azoles can be problematic (Alexander et al., 2007). Furthermore, like the ATB FUNGUS 3 method, this method still requires an incubation time of at least 24 h. Vitek 2 is a rapid detection system with significant automation advantages, capable of automatically inoculating, incubating, and reading data. However, the incubation time is relatively long, taking 14–18 h, with an average of about 15.5 h (Cuenca-Estrella et al., 2010). A common issue among these commercialization methods is the fixed types and concentrations of drugs, which prevents flexible combinations based on needs, and leads to inconveniences in applications. Matrix-assisted laser desorption ionization time of flight mass spectrometry (MALDI-TOF MS) is an innovative culture-based analytical technique. Marinach et al. utilized MALDI-TOF MS technology to detect the susceptibility of *C. albicans* to FCA, successfully shortening the culture time to 15 h (Marinach et al., 2009). Compared to the CLSI reference method, the CA was 88%, and the EA reached 100%. Vella et al. also utilized MALDI-TOF technology to assess the susceptibility of *C. albicans* to FCA, further shortening the culture time to 3 h (Delavy et al., 2020). However, when the culture time was significantly reduced, the EA compared to the EUCAST reference method was affected, dropping to only 86%. Therefore, the MALDI-TOF MS-based antifungal susceptibility testing method is still not ideal in terms of testing time. In addition, it is too complex to operate, hindering automation and requiring expensive equipment support. Flow cytometry is a culture-independent method that has achieved a significant breakthrough in detection time, capable of reducing the detection period to 2–8 h (Gokahmetoglu et al., 2003; Joung et al., 2007). However, it is difficult to select a single fluorescent dye suitable for testing with various commonly used antifungal drugs, which limits the throughput of the method. Moreover, this technique involves live fungi detection, posing a high biosafety risk. Additionally, the method requires expensive equipment, leading to high detection costs. Due to these various limitations, flow cytometry for antifungal susceptibility testing has not been adopted in clinical laboratories.

Fluorescent dyes are used in antifungal susceptibility testing to address the subjective issue of trailing drug interpretation because they are sensitive, selective, and cause minimal interference. Previous studies have also demonstrated that the fluorescent dye carboxyfluorescein diacetate (CFDA) and its modified versions can effectively assess the susceptibility of *Candida* to antifungal drugs when used in the standardized NCCLS M27-A BMD method (Liao et al., 2001; Liao et al., 2002). The fluorescent dye CFDA is added to the microdilution tray at the 24 or 48 h endpoint. While this method addresses the subjective issue of endpoint determination, it still requires a considerable amount of time.

Traditional fluorescent probes like CFDA commonly face the issue that fluorescence quenching or reduction occurs when fluorescent groups aggregate. However, the fluorescent small molecules used as probes often contain aromatic structures that are insoluble in water, thus they tend to aggregate in aqueous solutions; on the other hand, when they bind to biomolecules through physical or chemical interactions, these fluorescent molecules spontaneously aggregate. The result of aggregation is a significant decrease or even disappearance of the emitted fluorescent signal. Therefore, to avoid fluorescence quenching due to aggregation of the fluorescent probes, it is essential to strictly control the concentration of the probes and the number of probe molecules bound to biomolecules during use. This reduces the concentration of the fluorescent groups, leading to a significant decrease in the fluorescence intensity of the probes, and the sensitivity of detection remains not ideal.

To simultaneously address the issues of time-consuming procedures and subjective interpretation, we have developed a rapid and accurate quantitative method for *Candida* susceptibility testing based on AIE fluorescent molecule.

Tang et al. and Wang et al. each reported a fluorescence-based method for fast evaluation of bacterial susceptibility to antibiotics (Zhao et al., 2015; Zhu et al., 2011). Their research demonstrates that the fluorescence characteristics are closely aligned with the susceptibility of the bacteria tested. These findings clearly validate the feasibility of using AIE luminogens to evaluate bacterial susceptibility. Our team previously designed the AIE molecule DMASP, which successfully shortened the detection time for fungal susceptibility testing to just 12 h (Ge et al., 2022). To further accelerate this process, we have synthesized the AIE molecule TBP-2, which carries two positive charges, enabling a quicker assessment of *Candida* susceptibility to antifungal drugs. The cell wall of *Candida* consists of multiple layers of polyglycans, with the outer layer primarily composed of glucans and mannans, making the wall negatively charged (Gow et al., 2017). The cell membrane contains glycerophospholipids and sphingomyelin, further contributing to its overall negative charge. In contrast, TBP-2 contains two amine groups, making it positively charged (Shi et al., 2020a). The electrostatic interaction between TBP-2 and the negatively charged components of the cell wall and membrane plays a crucial role in *Candida* imaging. TBP-2 exists as individual molecules in solution, exhibiting faint fluorescence, due to its AIE properties. Upon encountering *Candida*, the positively charged TBP-2 molecules bind to the negatively charged cell wall and membrane through electrostatic interactions, leading to a pronounced increase in fluorescence. When antifungal drugs are absent or ineffective, *Candida* grows rapidly under suitable conditions, whereas the growth of *Candida* is partially or completely inhibited when antifungal drugs are effective. At the end of the *Candida* culture, TBP-2 is added to the culture medium, which emits light of varying intensity depending on the amount of *Candida* present. Since the fluorescence intensity of TBP-2 is linearly related to the concentration of *Candida*, the amount of *Candida* in these culture media containing different antibiotics can be easily identified by measuring the fluorescence intensity of these solutions, thus making it easy to determine the important parameter of antibiotic effectiveness, the MIC.

The standard strain *Candida* ATCC 90028 was used as a model to establish and validate the TBP-2-based method, with the MIC values falling within the expected range, confirming consistency with BMD method. To further validate the TBP-2-based detection system, we tested 76 strains of *C. albicans*, including 58 non-susceptible clinical isolates (resistant to at least one antifungal drug) and 18 susceptible strains. These clinical isolates covered a wide range of susceptibilities to FCA and VRC. The validation results showed excellent agreement (both EA and CA) with the BMD reference method, with no very major errors (VME) or major errors (ME) detected. Minor discrepancies, if any, were within the acceptable range specified by the CLSI M23-Ed6 document. These findings confirm the reliability of the TBP-2-based fluorescence method for antifungal drug screening. Notably, our method yields precise MIC values in less than 8.5 h, compared to the 24–48 h required for the BMD method. The TBP-2-based approach greatly shortens testing time and removes inaccuracies caused by subjective interpretation biases. This rapid turnaround is particularly crucial for immunocompromised patients, such as those with leukemia or bone marrow transplants, where timely and accurate results are essential for effective treatment.

In our study, we used an inoculation density of 1 × 10^6^ cells/mL, significantly higher than that used in BMD method. This higher inoculation density accelerates fungal growth, allowing for the MIC to be measured within 8 h. Additionally, TBP-2, a typical AIE molecule, can bind to a larger number of biomolecules compared to traditional fluorescent probes, resulting in stronger fluorescence intensity without the risk of fluorescence quenching due to aggregation, thus offering enhanced sensitivity. This makes fluorescent detection more convenient and accurate. The combination of highly sensitive fluorescent molecules and increased inoculation density is key to the success of our method, enabling fast and reliable detection. Notably, the TBP-2 molecule used in our study carries two positive charges, which enhances its binding capacity compared to molecules with only one charge, further improving detection sensitivity. Moreover, the fluorescent dye TBP-2 is applied post-incubation to the microdilution tray, ensuring it does not interfere with the complex interactions between *Candida* and the antifungal drugs.

In previous fluorescence-based tests for antifungal drug susceptibility, a nonlinear relationship between fluorescence intensity and fungal concentration has complicated the determination of parameters indicating drug effectiveness (Liao et al., 2001; Liao et al., 2002). However, in our study, we observed a strong linear correlation between fluorescence intensity changes and *Candida* concentration. This allows for easy identification of *Candida* levels in culture media containing different antifungal drugs by measuring the fluorescence intensity. From this, key parameters such as MIC can be accurately and easily determined.

Thanks to the AIE properties of TBP-2, unbound molecules remain non-emissive, with fluorescence activation occurring only when the molecules are bound to *Candida*. As a result, background fluorescence remains very low, even without the need for washing. This wash-free detection eliminates the risk of *Candida* loss during the washing process, simplifying the experimental procedure and increasing the accuracy of *Candida* quantification. The straightforward imaging process, enhanced accuracy, and large linear dynamic range make this method highly suitable for high-throughput antibiotic screening applications. Certainly, this study has some limitations. The number of susceptible and resistant strains should be more balanced. While our study included a broad range of MIC values for FCA and VRC, covering both susceptible and resistant strains, for AMB, only susceptible strains were included, and for 5-FC, only strains with MIC values ≤0.25 μg/mL were considered. This limitation is primarily due to the lack of isolated resistant strains at our institution. To provide a more comprehensive evaluation, future studies should expand the sample size and include a greater number of resistant strains.

## 5 Conclusion

In summary, we have developed a TBP-2-based antifungal susceptibility method, with data showing excellent agreement with the reference BMD method for the tested antifungals. This approach addresses the issues of long detection times and subjective endpoint interpretation common in traditional methods, offering rapid, objective, and quantifiable results that are easy to interpret. As a simple, fast, and precise quantitative detection technique, it enables the quick determination of accurate MIC values, supporting the timely selection of appropriate antifungal therapies and helping to reduce the emergence of resistant fungal strains. Furthermore, this method has the potential for automation and broad applicability.

## Data Availability

The raw data supporting the conclusions of this article will be made available by the authors, without undue reservation.
