# Supplementary material for: An AIE-active probe acts as novel tool for rapid and accurate quantitative antifungal susceptibility assessment
Source: Front Microbiol. 2025 Mar 27;16:1566846. doi: 10.3389/fmicb.2025.1566846 (PMC11983505; doi:10.3389/fmicb.2025.1566846)
Supplement: Supplementary file 2 [file Data_Sheet_1.docx]

**
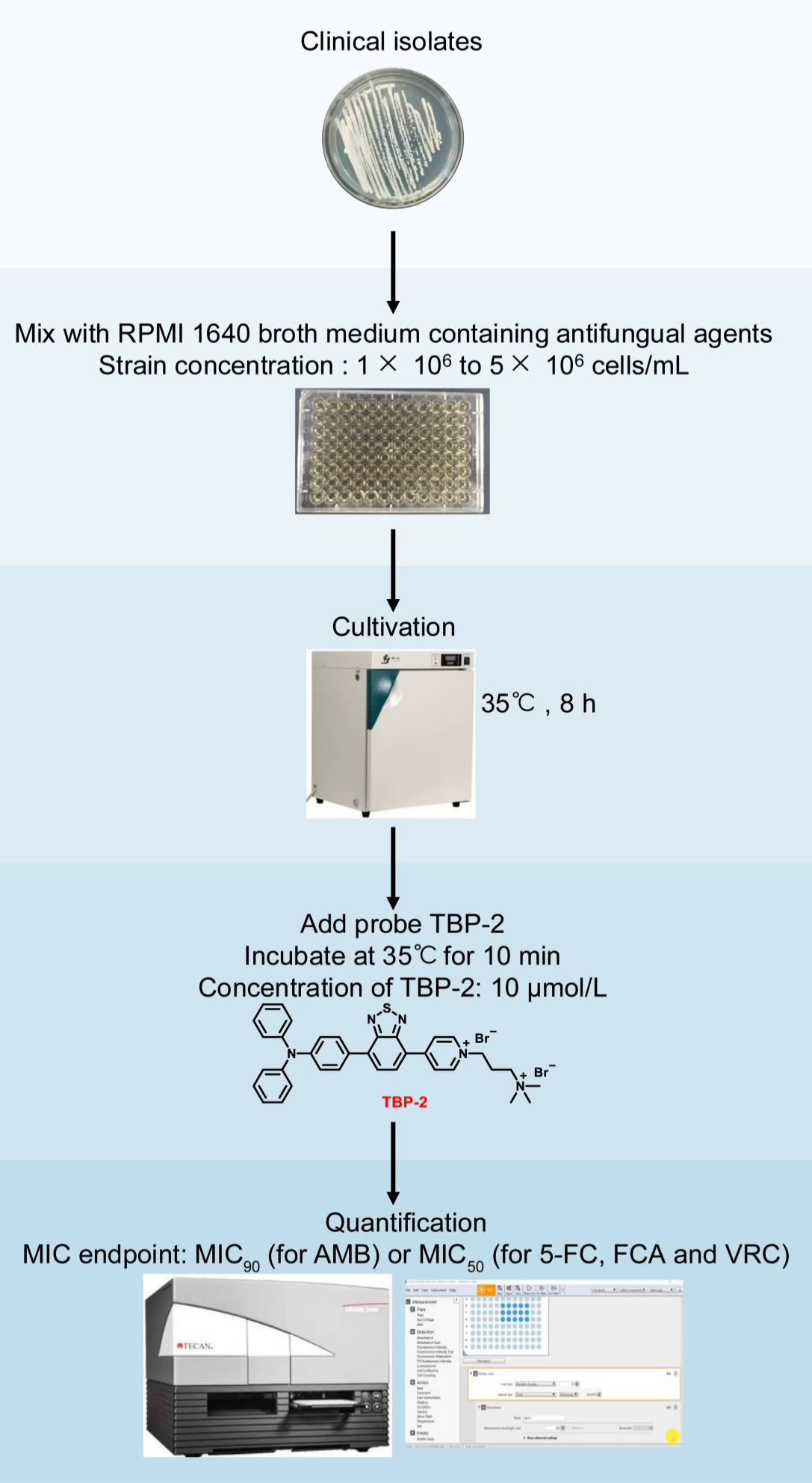
**

**Supplementary Figure S1**

Schematic illustration of the testing procedures of TBP-2-based method


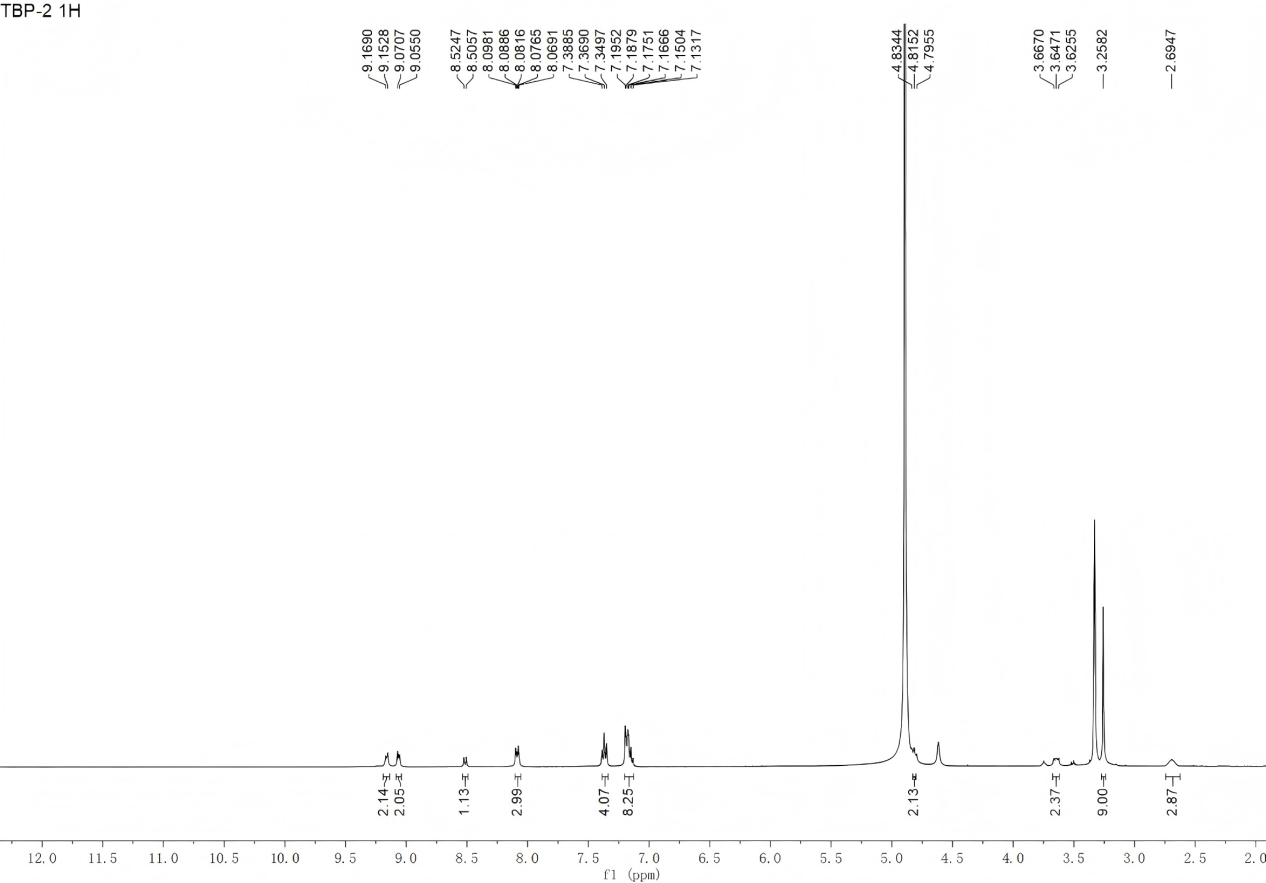


**Supplementary Figure S2**

^1^H NMR spectrum of TBP-2 in MeOD-d_4_.

**
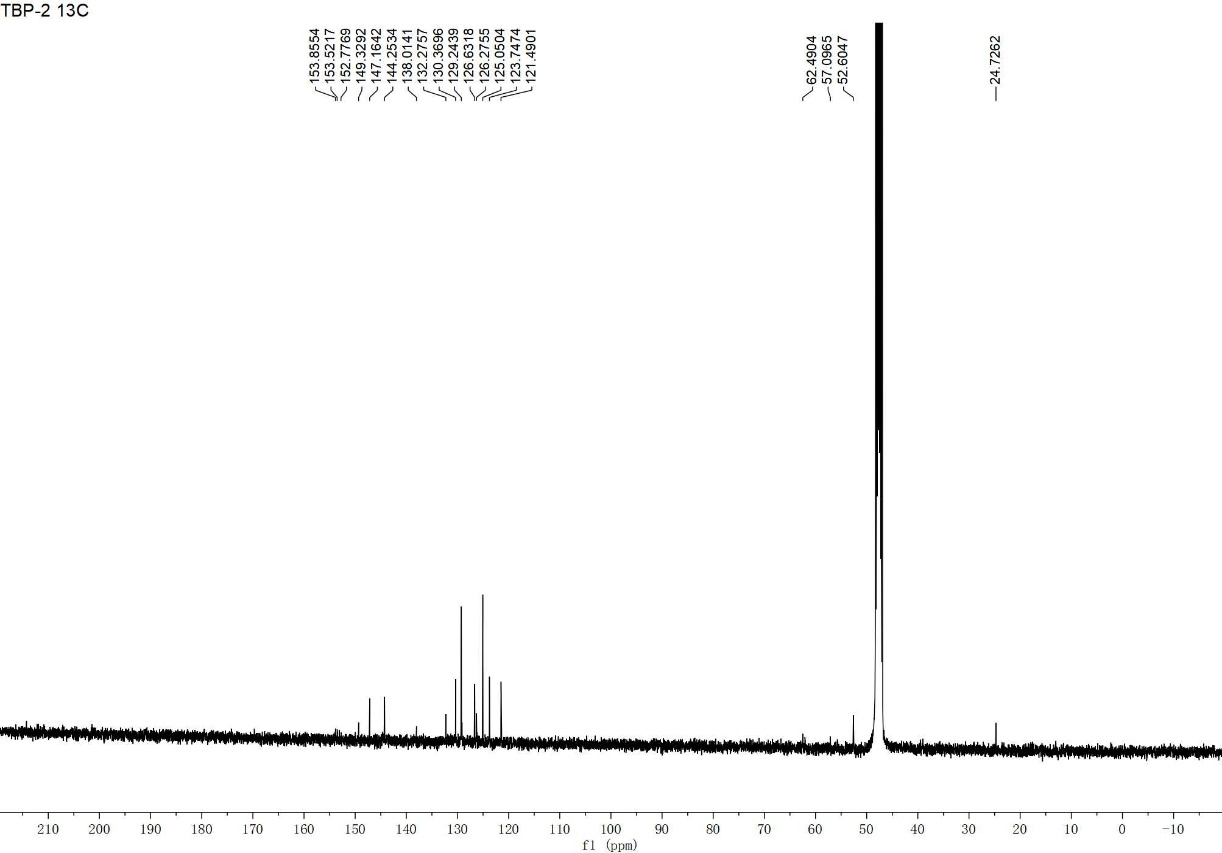
**

**Supplementary Figure S3**

^13^C NMR spectrum of TBP-2 in MeOD-d_4_

**
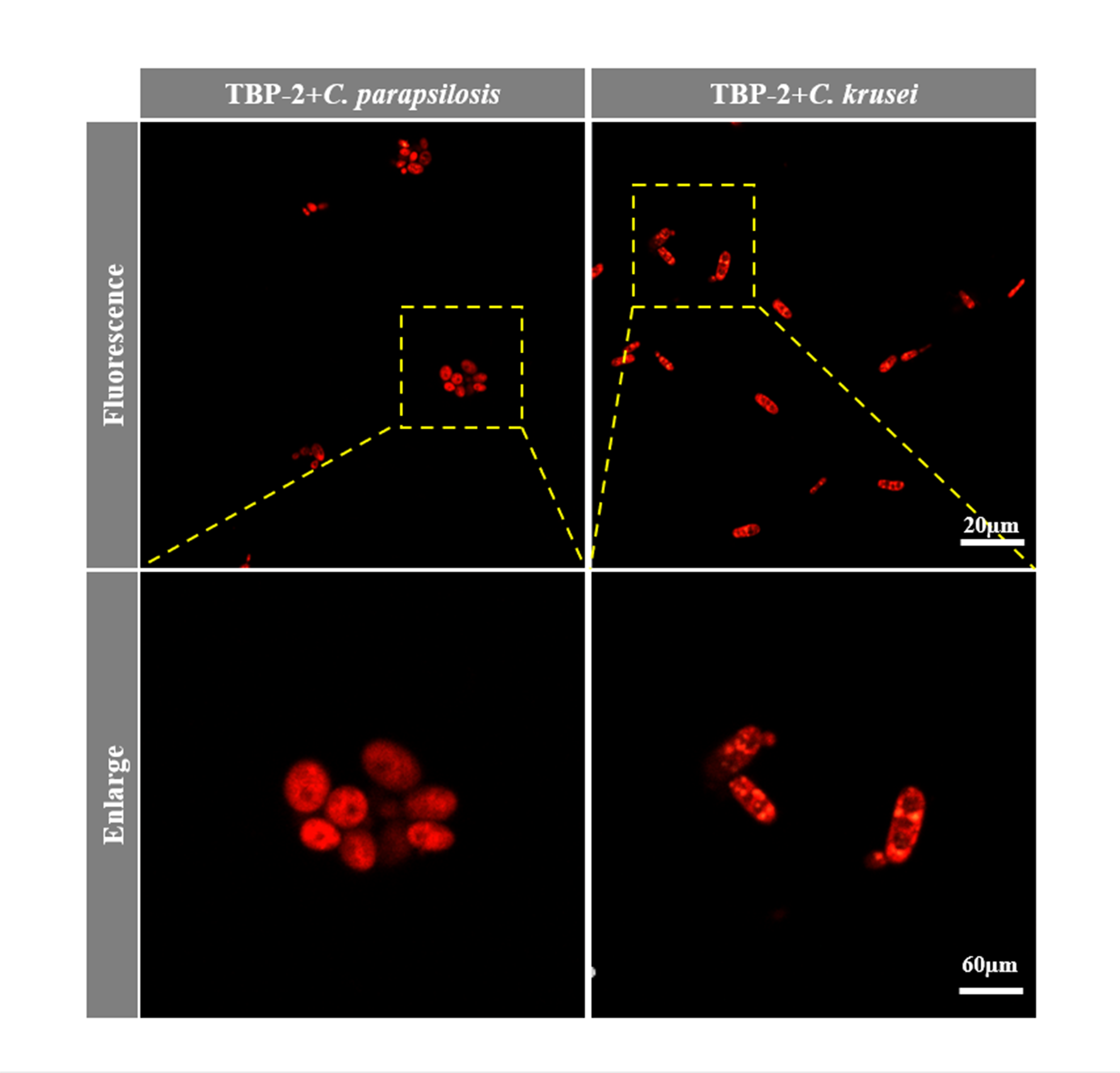
**

**Supplementary Figure S4**

Confocal laser scanning microscope (CLSM) image of *C.* *parapsilosis* and *C. krusei* incubated with 10μmol/L of TBP-2 for 10 min.

**Supplementary Table S1**

Changes in fluorescence intensity of TBP-2 in RPMI 1640 broth medium in the presence of different antifungal drugs.

| **Drug concentrations (μg/mL)** | **（*I*-*I_0_*）/*I_0_*** | | | |
| --- | --- | --- | --- | --- |
|  | **AMB** | **5-FC** | **FCA** | **VRC** |
| 150 | 0.05 | 0.02 | 0.02 | 0.04 |
| 128 | 0.10 | 0.03 | 0.04 | 0.05 |
| 64 | 0.08 | 0.09 | 0.03 | 0.01 |
| 32 | 0.08 | 0.06 | 0.07 | 0.06 |
| 16 | 0.03 | 0.01 | 0.01 | 0.07 |
| 8 | 0.06 | 0.05 | 0.02 | 0.02 |
| 4 | 0.01 | 0.07 | 0.06 | 0.07 |
| 2 | 0.08 | 0.03 | 0.10 | 0.09 |
| 1 | 0.10 | 0.06 | 0.09 | 0.03 |
| 0.5 | 0.04 | 0.03 | 0.03 | 0.03 |
| 0.25 | 0.01 | 0.04 | 0.03 | 0.07 |
| 0.12 | 0.02 | 0.02 | 0.09 | 0.10 |
| 0.06 | 0.06 | 0.05 | 0.00 | 0.03 |
| 0.03 | 0.10 | 0.03 | 0.03 | 0.07 |

*I_0_*: fluorescence intensity of RPMI 1640 broth medium. Condition: *λ*ex = 488 nm.
